# Differences in the Alcohol Preference Assessment of Shy and Bold Zebrafish

**DOI:** 10.3389/fnbeh.2022.810051

**Published:** 2022-02-24

**Authors:** Marina Sanson Bellot, Isabela Inforzato Guermandi, Bruno Camargo-dos-Santos, Percília Cardoso Giaquinto

**Affiliations:** ^1^Department of Structural and Functional Biology, Institute of Biosciences of Botucatu, São Paulo State University, Botucatu, São Paulo, Brazil; ^2^Aquaculture Center of Unesp, São Paulo State University, Jaboticabal, São Paulo, Brazil

**Keywords:** personality, zebrafish, reward, reinforcement, alcohol preference, comparative behavior, conditioned place preference, boldness

## Abstract

Individuals differ in their preference for alcohol and propensity to develop alcoholism, where the behavioral profile, such as the bold-shy axis, plays an important role for such a difference. However, literature is limited and conflicting on the causes and consequences of this relationship. Translational studies using animal models, such as zebrafish, can help identify behavioral traits that predispose individuals to drink alcohol compulsively. Here, the preference for alcohol was investigated in two distinct traits in zebrafish: shy and bold. For this purpose, fish were separated into shy and bold traits and then a conditioned place preference paradigm was used, a strategy that allows the rewarding effects from alcohol to be assessed by the ability to enhance the animal’s preference for an environment that initially was not preferred. It was found that bold zebrafish actively searched for the environment that was paired to alcohol after one acute exposure, whereas, shy fish changed their place preference even without alcohol administration, showing that the conditioned place preference protocol, given the short amount time to assess place preference, is not ample enough for shy fish to choose. Our results show that behavioral profiles must be considered in further studies since differences between shy and bold individuals on preference behavior can strongly interfere in the assessment of drug preference, mainly when using the conditioned place preference paradigm.

## Introduction

Individuals vary in their vulnerability to diseases and some behavioral profiles are more likely to develop them (Cavigelli, 2005). When dealing with substance use disorders, such as alcoholism, it is not fully understood why some people develop an addiction while others do not. It is widely understood to result from genetic/epigenetic and environmental factors, along with their interaction (Goldman et al., 2005; Ruggeri et al., 2015; Stacey et al., 2016), and individual differences in behavior plays a role in such variation (Skóra et al., 2020). For example, craving and relapse in alcohol dependents are related to certain personality traits, such as novelty-seeking and high impulsivity (Evren et al., 2012). A great deal of work has been done in gathering information on this relationship in animal models, such as rats and mice, to develop promising models to study human alcohol dependence (Radwanska and Kaczmarek, 2012; Belin-Rauscent et al., 2016; Montagud-Romero et al., 2016; Augier et al., 2018). Besides this, studies have shown that responses to new stimulus and psychoactive drugs are modulated by the same networks and neurotransmitters in synaptic connections (Dalley et al., 2007; Everitt et al., 2008; Wingo et al., 2016).

However, the main mechanisms behind the personality and drug addiction relationship are yet to clarified. The dilemma still exists of whether addiction itself leads to novelty-seeking and risk-taking behaviors or whether a personality traits showing these behaviors can be predisposed to drug use and dependence (Wingo et al., 2016; Skóra et al., 2020). Regarding alcohol abuse, there is conflicting evidence about the relationship between behavioral characteristics and alcohol consumption (Henniger et al., 2002; Nadal et al., 2002; Bahi, 2013; Momeni and Roman, 2014).

Animal models are used as the main strategy to investigate characteristics of this phenomenon. Fish may be an interesting alternative to investigate such an issue, allowing ancient aspects of our biology to be assessed. They provide a multiple species strategy approach, which allows mechanisms that are common among different taxon to be identified and focus on features that overlap between them, so aiming to find the core mechanism of a phenotypical trait (Gerlai, 2014). Until now, the impact of individual differences on fish drug preference and “seeking-like” behavior remains unknown. To the best of our knowledge, studies are restricted to how individuals with different exploratory profiles change their locomotor and social behavior after alcohol exposure, demonstrating that bold individuals are more resistant to the substance and change their behavior less than the shy ones (Araujo-Silva et al., 2018, Araujo-silva et al., 2020).

In pharmacological studies, zebrafish (*Danio rerio*), have emerged as a new animal model for understanding drug reinforcing properties. This species has high nucleotide homology with humans (Howe et al., 2013), neurotransmitters that are similar to mammalians—including the pharmacological receptors (Panula et al., 2006; Rico et al., 2011; Saleem and Kannan, 2018) showing considerable discoveries on the effects of drugs in animal behavior (Kedikian et al., 2013; Chacon and Luchiari, 2014; Faillace et al., 2018). Besides this, these fish present the shy and bold axis of personality, traits directly related with behaviors involved in personality predisposition to drugs, the novelty-seeking and risk-taking behaviors (Flagel et al., 2014). Bold fish are risk-takers and explorers (Øverli et al., 2006; Toms et al., 2010; Hulthén et al., 2017) and show less variation in behavior after environmental changes (Harcourt et al., 2010), while shy fish are usually neophobic and aversive to risks (Sneddon, 2003).

Investigating individual differences in fish drug “seeking-like” behavior through a conditioned place preference (CPP) may provide new data and insights regarding this phenomenon. The CPP assesses the rewarding effect of a drug by its ability to enhance the animal’s preference for an initially non-preferred environment (Mathur et al., 2011) and it is not known how individual differences in fish behavior influence this preference assessment. As such, in this study, the influence of bold and shy traits in zebrafish were investigated through the assessment of alcohol preference.

## Methods

### Animals and Experimental Conditions

Adult male and female of zebrafish were acquired from commercial fish farm and kept in 63 L tanks (1 fish/L). Tank water was kept at a temperature of 26–28°C, with ideal pH for the species (6.8–7.5) and low levels of ammonia (<0.02 mg/L) and nitrite (<0.1 mg/L). The photoperiod was 12/12—light/dark cycle, with constant aeration in the tanks and food with a commercial ration (35% protein).

### Experimental Design

Firstly, fish were separated by trait—shy or bold—and then their alcohol preference behavior was investigated. Since bold fish are novelty-seekers and risk-takers (Øverli et al., 2006; Toms et al., 2010; Hulthén et al., 2017) and shy fish are neophobic and aversive to risks (Sneddon, 2003) fish traits were set by evaluating their behavior in a new environment and in the presence of a predator, simulating a novel and a risky situation, respectively. Then, a CPP paradigm was used to assess both traits and alcohol preference. Four groups were established: (1) Bold treated with alcohol (*n* = 7); (2) Bold control (*n* = 9); (3) Shy treated with alcohol (*n* = 8); (4) Shy control (*n* = 8).

### Boldness Assessment

Fish were separated into shy and bold groups using “open-field” (Burns, 2008) and “predator” tests (Moretz et al., 2007), which evaluated the novelty-seeking behavior in fish in a new environment along with their risk-taking behavior in the presence of a predator. Two open-field tests and one predator test were performed for each individual, with 72 h intervals, allowing boldness to be assessed in 3 testing days and in 2 different contexts (open-field vs. predator).

Before boldness tests began, fish were individualized in the aquaria, so allowing them to be identified between tests. Open-field tests were performed in an aquarium (48 × 26 × 22 cm) with the bottom marked with 24 rectangles (5, 5 cm each). Fish were acclimatized for 1 min in the center of the aquarium inside a small and opaque cylinder, used only for habituation to water conditions. The animal was not able to swim or visualize the environment during habituation. Next, the number of lines crossed in 3 min was counted (Ariyomo and Watt, 2012) along with time of freezing behavior. Since zebrafish are a shoaling species, placing them alone in unfamiliar environments and assessing movement can indicate exploratory behavior (Ariyomo and Watt, 2012), while freezing indicates fear responses in novel environments (Kalueff et al., 2013), and both behaviors allow boldness to be assessed. The predator test consisted of placing a dummy model of a zebrafish predator, the *Etropeulus canarensis* (Moretz et al., 2007), at the end of each fish aquarium, and then measuring fish movement and freezing behavior for 5 min. Fish movement and freezing indicate both exploratory behavior and fear responses in risky situations, allowing boldness to be assessed. In this test, the aquarium was marked out with lines and fish movement was measured by the number of lines crossed. All the tests were recorded on video to assess the behaviors described above. After these tests had been completed, fish were ranked as shy or bold based on a boldness index (BI). Each index was composed using the behavior of only one fish during all 3 tests. The index was calculated using the following formula:


$$\mathrm{BI}=\log_{10}\left(\frac{\left(\mathrm{LC}1+\mathrm{LC}2+\mathrm{LCP}\right)}{\left(F1+F2+\mathrm{FP}+1\right)}+1\right)$$

Where:

LC1 = Lines crossed on the first open-field test.

LC2 = Lines crossed on the second open-field test.

LCP = Lines crossed on the predator test.

F1 = Freezing time on the first open-field test.

F2 = Freezing time on the second open-field test.

FP = Freezing time on the predator test.

BI reflects a score for boldness, i.e., the higher the index value, the bolder the fish. A logarithm was applied to normalize the data and the two “+1”s were added in the formula to avoid a 0 as a denominator and in the result (Camargo-dos-Santos et al., 2021). Fish that crossed a greater number of lines in the open-field and predator test (Ariyomo and Watt, 2012) with less time showing freezing behavior in both tests, consequently had higher BI scores. Fish that crossed fewer lines with more time showing freezing behavior had lower BI scores. Then, fish BI scores were ranked from highest to lowest and divided in quartiles: fish among the first quartile had the highest scores and were considered bold and those among the fourth quartile were considered shy. Fish with scores that fell in the second and the third quartile were categorized as intermediate. A total of 64 fish were tested in the open field and predator tests, and as such, the 16 fish from the first quartile were considered bold, 32 fish from the second and third quartile were considered intermediate and 16 fish from the fourth quartile were considered shy. Only shy and bold individuals were submitted to the following CPP test.

### Conditioned Place Preference

Approximately 1 week after boldness assessment, a CPP test was performed. This test aimed to assess shy and bold fish’s alcohol preference behavior. For each trait, two treatments were created: (1) Control-group, with only water, and (2) Treatment, with 1.0% of alcohol (99.5%, for analysis) (Collier et al., 2014) diluted in water. The CPP test assesses the rewarding effect of the alcohol by its ability to enhance the animal’s preference for an environment that was initially not preferred (biased CPP experimental design), also evaluating the preference of the fish for a place. The procedure was adapted from the CPP protocol described by Mathur et al. (2011).

**FIGURE 1:**
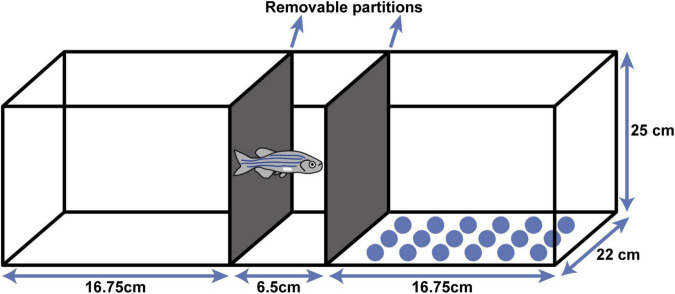
Conditioned place preference (CPP) aquarium. The aquarium was divided in three compartments using removable partitions—one in the center for acclimatization, one with a white bottom and one with blue circles on the bottom as visual cues.

The test was carried in an aquarium (40 × 22 × 25 cm) that had been divided into 3 compartments using removable partitions (Figure 1) and had been marked with different environmental cues: on one side the bottom of the tank had been covered with white paper and on the other, it had been covered with blue circles. Firstly, the initial preference of the fish was noted for one or other of the aquarium compartments. For acclimatization, each fish was placed individually in the central compartment of the aquarium for 2 min. Then, the partitions were removed, and the time spent in the white and dotted bottom compartments of the aquarium was recorded for 5 min. Due to extensive screening of the trait of the fish, initial preference analyses was optimized to reduce animal loss, using the following criteria: to determinate the initial preference of the fish for one compartment, they had to remain in one compartment for 60% or more of total time spent in compartments. This calculation only considered the targeting compartments (i.e., white and dotted, excluding time spent in central compartment).

Note that in all data analyzes, time spent in all compartments was considered, as per Mathur et al. (2011), including time spent in the central compartment. Only fish that presented these criteria performed the conditioning step. To ensure there was no preference bias for one compartment in particular (e.g., all fish had to present a preference for one side, however, the scenario where all individuals always prefer the same compartment was checked), all zebrafish were compared in their initial preference for the dotted vs. the white compartment, to ensure there was a balanced preference distribution between sides. In analyzing the data from the compartment bias test, time spent in the central compartment (Figure 1) was also considered.

The next day (24 h after the initial preference test) conditioning with alcohol was performed. The same aquarium was used for this step, but dividers were kept closed, so that the individual could not communicate or visualize the other compartments of the aquarium. Firstly, fish from the treated group were placed in the preferred compartment for 20 min only with water. Then, they were placed in the non-preferred compartment and exposed to 1% alcohol for 20 min. Thus, for example, if the individual initially preferred the compartment with the white bottom, initially, it was exposed to water in the compartment with white bottom and then exposed to alcohol in the compartment with the blue circles on the bottom, or vice-versa. Finally, fish were placed in an aquarium with only water for 2 min to wash the alcohol from their body. Fish from the control group followed the same procedure but received water instead of alcohol.

The last step consisted of evaluating the final preference of the fish for one compartment or the other and was performed 24 h after conditioning, using the same procedure as the initial preference. Therefore, the final preference of the fish was recorded without alcohol administration, with water only. Individuals stayed for 2 min in the central compartment and then the time spent in each compartment was recorded for 5 min (Mathur et al., 2011).

Besides an analyzes of how fish change their preference for a compartment that was initially not preferred, due to alcohol, the consistency of fish in preferring a place was also evaluated, measuring the percentage of time spent in the preferred compartment. This percentage was calculated in relation to the total time of the video recording, which included the time spent in both target compartments and the central compartment. Therefore, the percentage of time spent in the preferred compartment is not equally complementary to the time fish spent in the non-preferred compartment. For this reason, comparing the time fish spent in their preferred environment initially and the time spent in the same compartment after exposed to only water (i.e., control groups), can show how consistent the initial preference of the fish is for a certain place. As such, the time that bold and shy fish spent in the preferred compartment was analyzed in the first and the final step of the CPP procedure.

### Statistical Analysis

Before applying parametric tests, assumptions were tested using a Shapiro-Wilk test for normality and a Levene test for homoscedasticity. Then, for fish ranked as shy, intermediate and bold, the BIs of all quartiles (quartile 1, 2, 3, and 4) were compared using a one-way ANOVA. Fish from the first quartile were classified as bold, fish from the second and third quartiles were classified as intermediate and fish from the fourth quartile were classified as shy. To assess if fish presented a preference bias for one compartment in particular, the percentage of time spent in the white compartment vs. dotted compartment were compared using a *t*-test.

To evaluate the consistence in a place preference without alcohol exposure, a linear mixed-effect model was performed. In this model, “trait” (shy and bold) and “sampling time point” (initially and after water exposure) were set as fixed factors, and “fish” and “sex” as random factors. *Post hoc* comparisons using a Tukey HSD test were performed.

To analyze alcohol preference, four linear mixed-effect model were performed, one to each group separately: (1) shy control, (2) shy alcohol, (3) bold control, (4) bold alcohol. In these models, “sampling time point” (before and after conditioning) were set as fixed factors and “fish” and “sex” as random factors. Before applying all linear mixed-effect models, assumptions of linearity were checked, residuals homogeneity of variance, and normality of residuals. The significance level was set at α = 0.05 in all analyzes performed.

## Results

### Boldness Index

**FIGURE 2:**
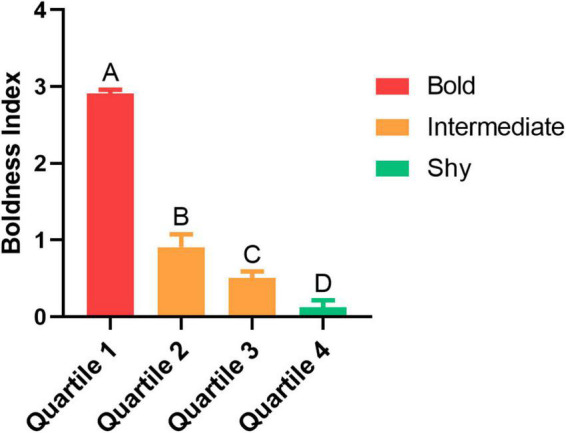
The boldness index (BI) score for each trait with their respective quartile and trait (bold, intermediate or shy). Different uppercase letters indicate significant difference between the quartiles (*n* = 16 per quartile; *p* < 0.05). Values are means ± SEM.

Fish were separated into shy and bold traits based on their BI score. The higher the index, the bolder the individual. Individuals classified with as “intermediate” were excluded from the experiment. All groups differed from each other (*p* < 0.001 for all comparisons, except Quartile 2 vs. Quartile 3, that *p* = 0.025; Figure 2). Only bold and shy fish were used to the following procedures (see Supplementary Table 1).

### Conditioned Place Preference

In this study, a CPP test was used to investigate the place and alcohol preference of fish. The place preference was evaluated by investigating the place preferred by the fish without alcohol administration, and for this, only the control groups were used. Alcohol preference was assessed by the amount of time fish spent in a compartment that initially was not preferred after receiving alcohol, that is, if the alcohol increased the preference of the fish for that place. The following results include: (1) Compartments bias test; (2) the preferred place of the fish; (3) alcohol preference of the fish.

#### Compartments Bias Test

The test aquarium had no preference bias, since, initially, zebrafish displayed no significant preference for the dotted vs. the white compartment (*t* = −0.563, df = 30.111, *p* = 0.578).

#### Preferred Place

**FIGURE 3:**
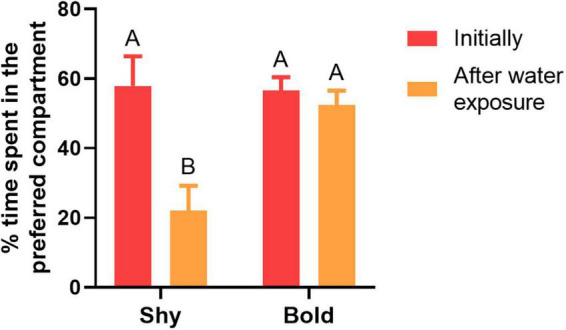
Shy (*n* = 8) and bold fish (*n* = 9) percentage of time spent in the preferred compartment initially and after water exposure. The percentage of preference is calculated based on the total time spent in the compartments, including the central compartment. The uppercase letters indicate significant differences between the treatments using the Tukey *post hoc* test (*p* < 0.05). Values are means ± SEM.

The following results compare the time that shy and bold fish, in the control group, initially spent in the preferred compartment vs. after water exposure only (Figure 3). There was significant difference in the percentage of time spent in the preferred compartment between the traits (*F* = 10.009, df = 36, *p* = 0.003), across the sampling time points (*F* = 16.827; df = 36; *p* < 0.001), and in the interaction between trait and sampling time points (*F* = 12.930; df = 36; *p* = 0.001). Shy fish spent less time in the preferred compartment after being exposed to only water (*p* < 0.001). On the other hand, bold individuals spent a similar time in the preferred compartment before and after being exposed to only water (*p* = 0.984; Figure 3).

#### Alcohol Preference

**FIGURE 4:**
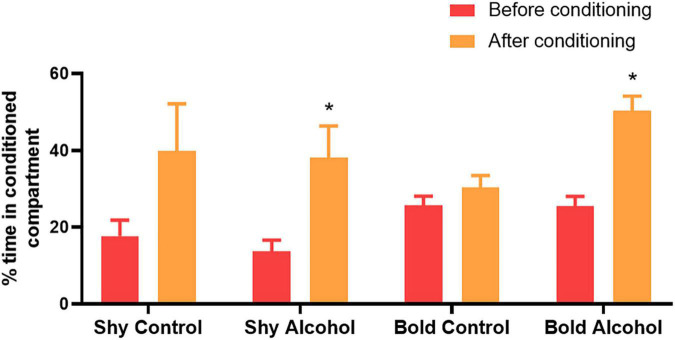
Percentage of time spent by shy or bold fish in the non-preferred compartment before and after only water exposure (control) or conditioning with 1% alcohol. The percentage of preference is calculated based on the total time spent in the compartments, including the central compartment. The asterisk indicates significant differences through the Tukey *post hoc* test between time spent before and after conditioning within each treatment (shy control, *n* = 8; shy alcohol, *n* = 8; bold control, *n* = 9, and bold alcohol, *n* = 7; *p* < 0.05). Values are means ± SEM.

The following results show the data regarding the time initially spent in the non-preferred compartment and after alcohol (treatments) or water exposure (control groups) (Figure 4). A significant difference was observed between the time spent in the non-preferred compartment before and after alcohol conditioning in the shy alcohol group (*p* < 0.001) and bold alcohol group (*p* = 0.007). Fish from these groups spent more time in non-preferred compartments after conditioning (Figure 4). Regarding the control groups, no significant difference was observed between time spent in the non-preferred compartment before and after water exposure in the shy control group (*p* = 0.078) and in the bold control group (*p* = 0.749). However, it is important to highlight that shy control with treatment spent twofold more time in the conditioned compartment when exposed to only water, even thought it was not statistically significant (*p* = 0.078).

## Discussion

In this study, the influence of bold and shy behavioral traits in zebrafish were investigated on the assessment of alcohol preference. Overall, individual differences in behavior were shown that can affect the performance of zebrafish in the CPP test, and that fish trait must be considered in studies that investigate alcohol preference using this methodological strategy. Bold individuals treated with alcohol actively searched for the alcohol related environment and had an increase in preference for the conditioned chamber by ∼25%, meanwhile bold fish from the control group spent similar time in the environment initially preferred after water exposure in the non-preferred compartment and had an increase in preference by only ∼4.55% for this compartment. This showed that bold zebrafish changed the place that they initially preferred due to alcohol. Shy fish treated with alcohol also spent more time in the alcohol related environment, with an increase in preference by ∼24.54% (Figure 4). However, the shy control group also spent twofold more time in the conditioned compartment when exposed to water, with an increase in preference by ∼22.29%, thus decreasing preference for the initially preferred compartment by up to ∼35.83%, changing place preference without alcohol stimuli (Figure 3). These results suggest that, since shy fish exhibit a place preference in response to water and alcohol, this response is independent of alcohol. However, the possibility that shy fish presented CPP related to alcohol cannot be excluded, since they spent more time in the conditioned environment after alcohol exposure.

These results indicate that shy characteristics *per se* interfered in the alcohol preference assessment. Possibly, because they are more cautious in unfamiliar situations (Toms et al., 2010), they may take longer periods of time to establish place preferences. Besides this, shy fish demonstrate greater ranges of reactions when facing choice and have less patterned behavioral responses to the environment (Harcourt et al., 2010; Jolles et al., 2019), indicating that having a shy trait can influence preference assessment. It is important to notice that when individuals are unresponsive or do not behave in determined criteria in preference studies, it is usually considered experimental error (Pruitt et al., 2011). In several studies, fish with a strong initial preference for one place were excluded from the study (Darland and Dowling, 2001; Kily et al., 2008; Mathur et al., 2011; Montagud-Romero et al., 2016). Analyzing the results from the present study of initial preference between bold and shy fish, several shy individuals had a strong initial preference for one side (Figure 3). Therefore, shy fish may probably be excluded from the afore mentioned studies. Also, note that individual variation in preference responses are commonly underestimated (Campbell and Hauber, 2009; Pruitt et al., 2011). The CPP test is particularly one of the most common and popular models to study drug rewarding effects (Tzschentke, 2007) but it has not been used to investigate how individual variation can impact the drug preference evaluation, as shown by our results.

Regarding bold individuals, they spent a similar amount of time in their preferred compartment before and after water exposure in the non-preferred side, as such, not changing their initial preference. Also, they spent more time in the conditioned compartment after receiving alcohol while the control group did not increase its preference for the non-preferred compartment. These findings show that bold individuals choose a place preference faster than shy fish and show preference for alcohol after one acute exposure. Therefore, the CPP test with a short period strategy for place preference evaluation, as proposed in Mathur et al. (2011) CPP protocol is suitable for testing alcohol preference in this personality trait, but not for the shy trait. Since bold individuals are explorers and risk-takers (Toms et al., 2010; Hulthén et al., 2017), it is reasonable to use a new environment for assessing preference.

Considering time to choose as a factor to discriminate choosier and less choosy individuals, bold individuals may be less choosy, choosing a place preference faster. On the other hand, shy fish may be choosier, probably taking longer periods of time to prefer one environment. This could indicate that the bold-shy axis is related to the choosiness trait, which is defined as the strength of preference for some options over the others (Sih and Bell, 2008). Several studies indicate that some traits are interrelated, e.g., boldness, aggressiveness, and activity, due correlated selection (Dingemanse et al., 2007) or shared proximate mechanisms (Koolhaas et al., 2010; Kralj-Fišer et al., 2010).

We understand that this is the first time that evidence has been shown of a possible relationship between the bold-shy axis of personality and the choosiness trait. These findings can have implications in personalities studies, since comprehending how traits are related is important to understand how they evolved and were maintained (Pruitt et al., 2011). However, as behaviors analyzed in this study were evaluated in short periods of time (between weeks), it is not possible to state that shy and bold fish would maintain this pattern of behavior across different ontogenetic stages. Thus, further studies are needed to provide more evidence regarding the relationship between the shy and bold axis and the choosiness trait, by assessing this relationship over longer periods of time.

Besides this, our findings on bold behavior also bring new insights regarding the CPP strategies which ensured individuals had no initial preference in order to be conditioned in one place (Shimosato and Watanabe, 2003; Arenas et al., 2014; Montagud-Romero et al., 2016). Possibly, as they tried to prevent a methodological bias, they might have created another by excluding the bold personality from the study, since this trait responds quickly to the environment, choosing a side quickly and having an initial preference.

Increasingly, zebrafish are gaining more space to be used as an animal model in translational studies, since individuals not only present behavioral and neurological responses to widely consumed drugs, such as alcohol (Rico et al., 2011; Chacon and Luchiari, 2014), but they also show evidence of the relationship between individual differences in behavior and the effects of alcohol (Araujo-Silva et al., 2018, Araujo-silva et al., 2020), a complex phenomenon to study in humans. The findings that individual differences can, as showed in this study, influence the assessment of alcohol preference, may contribute to developing better strategies to investigate the relationship between behavioral profiles and alcohol in zebrafish. Investigating these issues in laboratory animals may provide the identification of behavioral traits that predispose individuals to compulsively drink alcohol and could allow neural and molecular substrates of alcoholism to be found, thus enabling new outcomes of possible therapies (Skóra et al., 2020).

Overall, our findings can help researchers not only prepare the study methodology but also to understand how differences between shy and bold individuals on preference behavior can strongly interfere in drug preference evaluation, mainly when using the CPP paradigm. Studying extreme personalities can indicate mechanisms within a population, which could often be hidden by analyzing more frequent behaviors. The results indicate there is no better or worse performance between shy and bold individuals, but each profile exhibits a different way to establish preference in classical preference test used in pharmacological studies, such as CPP. Shy and bold fish present different needs when tested and used as a model to study the effects of drugs on behavior. Ultimately, this information may be useful for research seeking ways to treat addiction, since tests in preclinical trials could provide answers for a particular group or behavioral trait, excluding the characteristics of other behavioral profiles that could also help in the development of better treatments.

## Data Availability Statement

The raw data supporting the conclusions of this article will be made available by the authors, without undue reservation.

## Ethics Statement

The animal study was reviewed and approved by the Committee on Ethics in the Use of Animals of São Paulo State University—CEUA protocol #1144.

## Author Contributions

MB, BC-d-S, IG, and PG contributed to conception and design of the study. MB and BC-d-S organized the database. BC-d-S performed the statistical analysis. MB and IG wrote the first draft of the manuscript. MB, BC-d-S, and IG wrote sections of the manuscript. All authors contributed to manuscript revision, read, and approved the submitted version.

## Conflict of Interest

The authors declare that the research was conducted in the absence of any commercial or financial relationships that could be construed as a potential conflict of interest.

## Publisher’s Note

All claims expressed in this article are solely those of the authors and do not necessarily represent those of their affiliated organizations, or those of the publisher, the editors and the reviewers. Any product that may be evaluated in this article, or claim that may be made by its manufacturer, is not guaranteed or endorsed by the publisher.
